# Green Processes for Green Products: The Use of Supercritical CO_2_ as Green Solvent for Compatibilized Polymer Blends

**DOI:** 10.3390/polym10111285

**Published:** 2018-11-19

**Authors:** Muhammad Iqbal, Christiaan Mensen, Xiaohua Qian, Francesco Picchioni

**Affiliations:** 1 Department of Chemistry, Institut Teknologi Bandung, Jalan Ganesha No. 10, Bandung 40132, Indonesia; iqbal@chem.itb.ac.id; 2 Engineering and Technology Institute Groningen (ENTEG), Chemical Product Engineering, University of Groningen, Nijenborgh 4, 9747 AG Groningen, The Netherlands; christiaanmensen@hotmail.com (C.M.); Sunny.Wit-Qian-de@dsm.com (X.Q.)

**Keywords:** starch, polycaprolactone, glycidyl methacrylate, compatibilization, supercritical carbon dioxide, mechanical properties

## Abstract

Polycaprolactone-*g*-glycidyl methacrylate (PCL-*g*-GMA), a reactive interfacial agent for PCL-starch blends, is synthesized using supercritical carbon dioxide (scCO_2_) as reaction medium and relatively high molecular weight PCL (*M*_w_ = 50,000). Higher GMA and radical initiator intakes lead to higher functionalization degrees (FD) for PCL-*g*-GMA samples. A mathematical model is developed to describe the correlation between monomer and initiator intake and FD values. The model shows an excellent R^2^-value (0.978), which implies a good fit of the experimental data. Comparison of this model with a similar one for the reaction in the melt clearly indicates a better reaction efficiency in scCO_2_. Furthermore, GPC results show that less degradation occurred for samples made in scCO_2_. Finally, the use of the PCL-*g*-GMA made in scCO_2_ (as interfacial agent) in ternary blend of PCL/starch/PCL-*g*-GMA results in better mechanical properties with respect to those obtained by using the same graft-copolymer as prepared in the melt.

## 1. Introduction

Plastic products, with a global production of approximately 335 million tons annual and an annual growth of approximately 2.5% (in 2016), represent the largest field of application for crude oil outside the energy and transportation sectors. The plastic industry is so dependent on oil that if the price of crude oil increases, a negative effect on the plastic market follows as consequence. Another important issue is the accumulation of plastic waste that is not easily degraded [1]. A lot of research has been performed to overcome these problems; the general field of “bioplastics” being in this case a paradigmatic example.

Starch is a biopolymer already widely researched for application in bioplastics. However, direct use of starch usually results in rather poor mechanical properties (e.g., tensile strength) and in relevant drawbacks coupled with the processability of the corresponding products [2]. A popular approach to solve these problems involves the blending of starch with a synthetic polymer such as polycaprolactone (PCL). The blend is expected to have good mechanical properties while maintaining its biodegradability [3,4]. However, phase separation takes place and a coarse morphology is obtained when simply blending starch with PCL [3,5]. In this context, the use of an interfacial agent represents a popular choice in order to improve the adhesion at the interface between the two polymers [4,6,7,8,9,10].

Generally, interfacial agents based on chemically modified PCL itself have been used to improve the system. The interfacial agent can be synthesized by functionalizing PCL with different monomers (functional groups). Another option is to graft PCL to starch by in situ ring-opening polymerization of ε-caprolactone in the presence of starch particles and using a catalyst [4,7,8]. The latter route is considered more complex than the functionalization of PCL. Monomers such as GMA [9,11,12,13], maleic anhydride (MAH) [9,10,14,15,16], pyromellitic anhydride [6], diethyl maleate (DEM) [13], and acrylic acid (AA) [10] have been successfully grafted onto PCL backbone for compatibilization purposes. These researches also concluded that functionalization with GMA results in the highest efficiency. In general, the grafting reaction of GMA is performed in a melt state due to its simplicity. However, grafting reaction carried out in melt would also result in unwanted side-reactions, such as PCL degradation. This problem can be solved by using supercritical carbon dioxide (scCO_2_) as grafting medium. Moreover, due to the plasticizing effect of scCO_2_ a melting point depression is observed and thus a lower processing temperature can be applied. The supercritical fluid is also thought to act as “transporting agent”, thus improving mass transfer during the mixing and leading to higher conversion [17]. Several monomers such as GMA [18], MAH [19], methyl acrylate [20,21], and methyl methacrylate [22] have been already and successfully grafted onto polypropylene using supercritical carbon dioxide as reaction medium. However, a systematic study linking the chemical composition of the feed to the reaction course (e.g., to the obtained FD values) is still missing in the open literature. To the best of our knowledge, also the use of scCO_2_ as grafting medium for polyesters (PCL in this case) has been not yet described, even if spectroscopic evidence clearly suggests relevant solubility of scCO_2_ in this kind of polymers [23].

This research focuses on the development of interfacial agents (PCL-*g*-GMA from high molecular weight PCL) using scCO_2_ as grafting medium. The obtained graft copolymers are then used in PCL/starch blends and their influence on the mechanical properties established. In both cases (functionalization and blending), the corresponding melt-based processes/products will be taken as reference to establish the influence, if any, of scCO_2_ on the reaction efficiency and the final product properties.

## 2. Materials and Methods

### 2.1. Materials

Polycaprolactone (PCL, CAPA 6503, *M*_w_ = 50,000) from Solvay Caprolactones, Warrington, UK, was used for the blends and the preparation of the interfacial agents without further purification or drying. Glycidyl methacrylate (GMA, purity of 97%, Aldrich, Zwijndrecht, The Netherlands) as grafting monomer was also used without further purification. Azobisisobutyronitrile (AIBN, purity of 98%, Acros Organics, Landsmeer, The Netherlands) and benzoyl peroxide (BPO, 75% purity, from Merck, Darmstadt, Germany) were used as radical initiators. Carbon dioxide (Linde Schiedam, CO_2_) and nitrogen (Linde Schiedam, N_2_) were used during the grafting reaction. Tetrahydrofuran (THF, >99% purity from Acros, Geel, Belgium) and methanol (99.8% purity from Labscan, Dublin, Ireland) were used during the purification process. Potato starch (with 75% amylopectin and 25% amylose) from Avebe, Veendam, The Netherlands, was used in PCL/starch blends after being dried under vacuum for 24 h (110 °C).

### 2.2. Synthesis and Purification of PCL-g-GMA

*Melt grafting*. The reactive compatibilizers were prepared in a Brabender (Plasticorder PL2000) batch kneader with a chamber volume of 35 cm^3^. The intake volume of reagents was set on 24 cm^3^, which is about 69% of the chamber volume to ensure a proper mixing. The kneader was heated to 130 °C with a rotation speed of 80 rpm. PCL was added when the required temperature was reached. A solution of BPO in GMA was added drop by drop over a period of 5 min. The materials were mixed for another 5 min, before the chamber was opened to collect the samples. An overview of the prepared samples is provided in Table S1 (Supplementary Materials).

**Figure 1:**
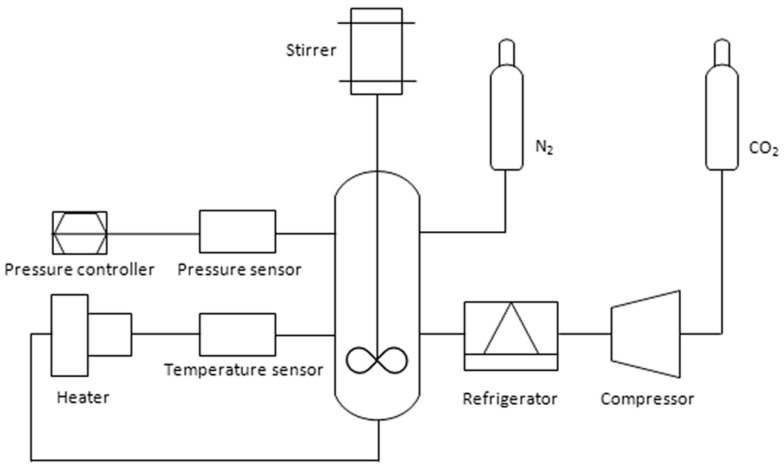
Experimental setup scheme for synthesis of PCL-*g*-GMA in scCO_2._

*Grafting in scCO_2_*. The interfacial agent was prepared in a Parr reactor (chamber volume 100 cm^3^) equipped with heating mantle and turbine impeller (Figure 1). A compressor (LEWA) was used to bring the reactor to the desired pressure. PCL, initiator, and monomer were weighted and placed in the reactor. Intakes and results for each experiment are given in Table 1. The reactor was flushed with nitrogen (10–30 bars) for 10 min to remove oxygen from the system. After that, the nitrogen was released, and the CO_2_ valve was opened to pressurize the reactor to approximately 50 bars. The heating mantle was set to the desired temperature and when this was reached the reactor was pressurized to the desired value. A stirrer (operated at 900 rpm) was used to achieve better mixing. The reaction started when the desired temperature and pressure were reached. After a given reaction time, the stirrer and the heating mantle were turned off and the valve was opened to depressurize the reactor. The reactor was opened to collect the samples, which were immediately quenched in liquid nitrogen. 

#### 2.2.1. Purification of PCL-*g*-GMA

To remove the unreacted GMA monomer, decomposed BPO, and GMA homo-polymers, a further purification of PCL-*g*-GMA is necessary [13]. PCL-*g*-GMA (5 g) was dissolved and stirred for about 1.5–2 h in THF (100 mL). Methanol (400 mL) was then added for precipitation at 6–8 °C (overnight). The suspension was decanted and the solid product was dried in a vacuum oven (40 °C, 5 mbar) until constant weight [13].

#### 2.2.2. Ternary Blend of PCL/Starch/PCL-*g*-GMA

The blends were prepared in a Brabender (Plasticorder PL2000) batch kneader (chamber volume 35 cm^3^). The intake volume was set on 24 cm^3^, to ensure a proper mixing. The kneader was heated to 170 °C with a rotation speed of 80 rpm. PCL, starch, and PCL-*g*-GMA were premixed in a beaker before being added to the mixing chamber. The mixture was blended properly for 15 min followed by sample collection [9,13].

### 2.3. Analytical Methods

#### 2.3.1. ^1^H-NMR

To characterize the reactive interfacial agent samples, ^1^H-NMR measurements were performed. The ^1^H-NMR spectra were obtained by using a 400 MHz Varian AMX Oxford NMR apparatus with CDCl_3_ (99.8%, Aldrich) as the solvent.

The FD could be calculated from the following:

(1) $$\;FD\left(\%\right)=\frac{A_{monomer}}{A_{polymer}}\times 100\;$$

The amount of moles of GMA attached to the PCL backbone was calculated by dividing the area of the peak of a characteristic proton belonging to the GMA (–CH< proton of the epoxide ring at δ 3.2 ppm, *A_monomer_*) by that of a characteristic proton of the PCL backbone (–CH_2_– protons at δ 4.0 ppm, *A_polymer_*) [13,15,24]. Furthermore, the efficiency of the grafting process, E, is defined by comparing the value of *FD* (experimental) with maximum obtained FD (theoretically based on the feed composition) as follows:

(2) $$\;\;E\left(\%\right)=\frac{FD}{FD_{max}}\times 100\;$$

#### 2.3.2. Gel Permeation Chromatography

Gel permeation chromatography was used for molecular weight and polydispersity (PDI) determination. The samples were dissolved in THF (10 mg/mL) with toluene as flow marker. The analysis was performed on a Hewlett-Packard 1100 system equipped with three PL-gel 3 μm MIXED-E columns in series. The column was operated at 42 °C with eluent flow rate of 1.0 mL/min, and a GBC LC 1240 RI detector was equipped. The average molecular weight was calculated using a calibration curve from two known PCL samples.

#### 2.3.3. Tensile Tests

To characterize the mechanical properties of the samples, tensile test was performed by using an Instron 4301 pulling bench in accordance with ASTM D1708. The dumbbell-shaped microtensile specimens were prepared by using a Fontijne Holland (TH 400) hot press machine. From one sequence of pressing, eight specimens (17.5 mm length, 4.4 mm width and 2.0 mm thickness) were obtained. The press temperature was 150 °C and a force of 150 KN was applied for 3 min. A water flow of 30% was applied to cool down the mold until reach room temperature, while the pressure was maintained. For every specimen, strain at break, stress at break, and elastic modulus were measured with a pulling rate of 50 mm/min. The corresponding value for every blend was calculated from an average of six measurements while the standard deviation was taken as the absolute error of the average values.

#### 2.3.4. Scanning Electronic Microscopy (SEM)

SEM was performed by using a Jeol 6320 F Scanning Electron Microscope. Before analysis, the samples were covered with a palladium/platinum conductive layer of 20 nm thickness, created by using a Cressington 208 sputter coater.

#### 2.3.5. Selective Solvent Extraction

To characterize, albeit indirectly, the chemical reaction at the interface between the –OH groups on the starch and the epoxide ones on PCL-*g*-GMA, we performed a selective solvent extraction in chloroform. 3 g of finely grounded samples were then extracted at room temperature for 48 h in 250 mL chloroform. The remaining solid (insoluble fraction) was then filtrated while the solvent was evaporated from soluble one. Both fractions were then dried in a vacuum oven and weighted. As PCL and PCL-*g*-GMA are fully soluble in chloroform under these conditions, an amount of insoluble fraction higher than the corresponding starch content indicates the presence of PCL coupled to the starch phase.

#### 2.3.6. Differential Scanning Calorimetry (DSC)

DSC measurements were performed by using a Q1000 TA instrument equipped with a TA instrument DSC cooling system. Each sample was initially heated from 0 to 100 °C (heating rate 10 °C/min) to remove the thermal history of the material. The transition temperatures of each sample were further determined by firstly cooling down the samples from 100 to 0 °C and subsequently heating up back to 100 °C (cooling and heating rate were 10 °C/min).

## 3. Results

### 3.1. Functionalization Reaction of PCL-g-GMA

Sixteen different graft copolymers (PCL-*g*-GMA) were prepared by reacting GMA with PCL (*M*_w_ 50,000) using AIBN as radical initiator. The obtained FD and E values are based on the calculation from NMR analysis (Table 1). 

**Figure 2:**
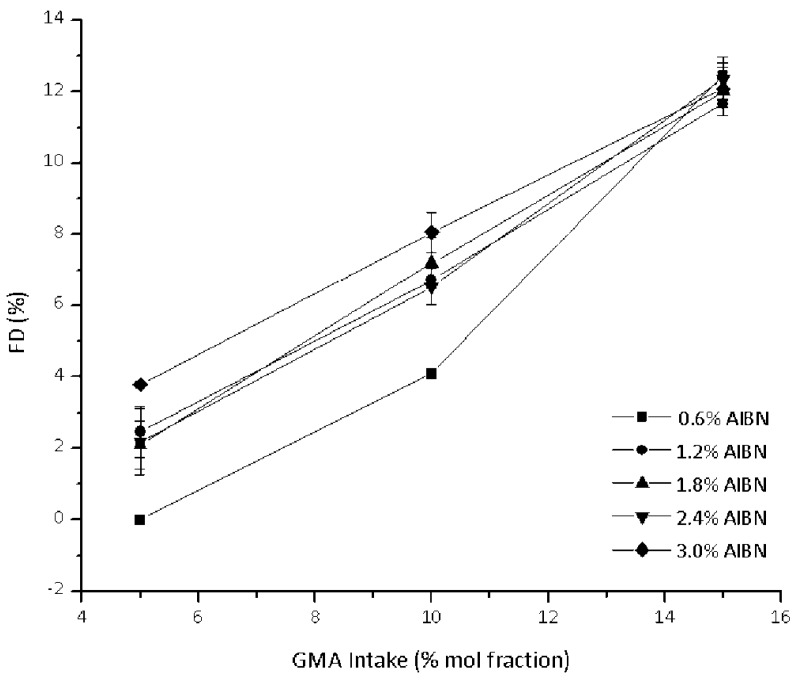
Effect of GMA intake on the FD values of PCL-*g*-GMA.

For comparison purposes, a similar overview of experiments carried out in the melt is provided in Supplementary Materials (Table S1). The obtained results show that higher GMA intakes lead to higher FD values (Figure 2). Similar results are observed for GMA grafting onto PCL in the melt even though using another radical initiator [11,13,25]. This trend is commonly explained by considering the higher GMA concentration, thus leading to faster grafting reaction rate. In addition, GMA is usually present as homopolymer grafted onto the PCL backbone [9,11].

**Figure 3:**
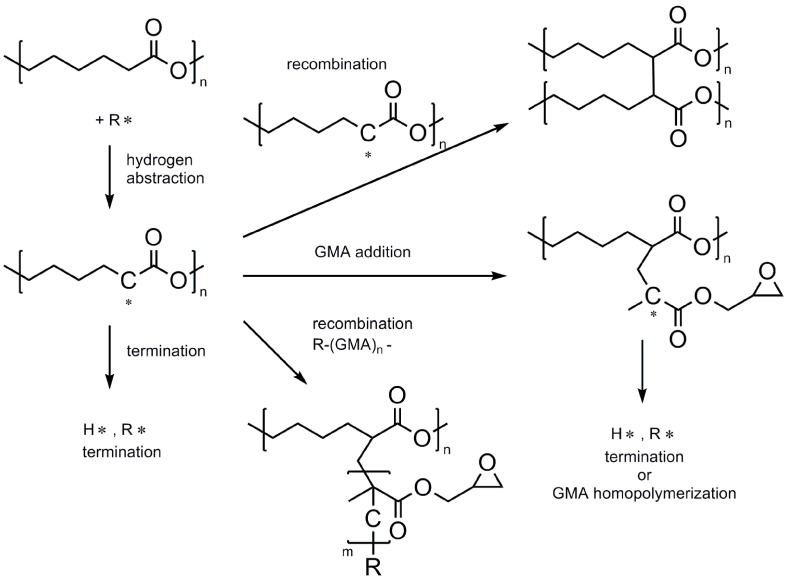
Simplified reaction pathway for grafting of GMA to PCL [9,11].

This homo-propagation reaction (of the first GMA molecule grafted on the PCL) is usually invoked to explain the relatively high GMA consumption rate and FD values [11]. In our case, the increase in FD is only slightly affected (Figure 2) by the AIBN amount except at the lowest employed intake (0.6%). A possible explanation for this behavior is probably connected to the reaction mechanism, as already described in the literature and reported in Figure 3 [9,11]. After the hydrogen abstraction from the PCL backbone by the primary radicals generated from the initiator, a PCL macro-radical is formed. This has four available reaction pathways: it can give coupling with low molecular weight radicals present in the system (resulting in a termination step), with a growing GMA chain (yielding a grafted product) or with another macro-radical (yielding a crosslinked product). Furthermore, it can also give addition to the GMA monomer, which can in turn propagate to yield the desired graft-copolymer or give H-abstraction (not shown for brevity). Two parallel reaction pathways (i.e., grafting of a single GMA molecule followed by homopolymerization or GMA homopolymerization followed by coupling with a macro-radical) yield in principle the desired product. However, the first is clearly favored from a statistical point of view since the chance for recombination of a GMA growing chain with a macro-radical (in principle a second order kinetic process) is supposedly smaller than the one for the addition of a GMA molecule to the same macro-radical. Degradation reactions (not shown for brevity) might also take place [13,15].

**Figure 4:**
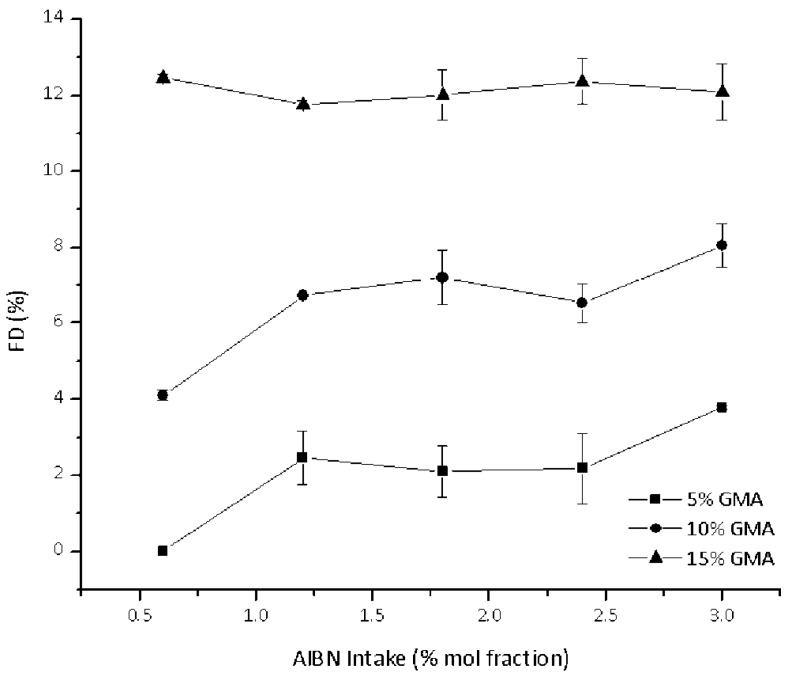
Effect of AIBN intake on the FD values of PCL-*g*-GMA.

At low monomer intakes (5% and 10%), the FD degree increases with the amount of initiator used (Figure 4). This can be easily explained by making allowances for the increased number of radicals generated at high AIBN intakes, which in turn can easily give addition to GMA. The trend is not monotonous, but the FD seems to level off (or only slightly increase) for AIBN intakes larger than 1.5 mol %). In this case, cage-effects can be invoked to explain this trend [26].

For highest monomer intake (15%), the FD can be considered as constant and independent of the initiator amount. To the best of our knowledge, this has not been previously observed for functionalization reactions in scCO_2_. One might speculate that the relatively high concentration of GMA in a radical-rich environment might lead to GMA homopolymerization with only a slight detectable effect on the FD ratio. This is not surprising if one considers the relatively low chance for GMA homopolymer recombination with a macro-radical (*vide supra*).

The discussion above clearly highlights the synergistic effect of monomer and initiator intake in determining the final FD. The combined influence can be conveniently described by using a multivariable regression procedure. The obtained model (Equation (3)) assumes that FD = *f*(*n_m_*, *n_i_*), where n_m_ is the amount of GMA and n_i_ is the amount of AIBN in the feed.

(3) $$\;FD=-5.424-2.939n_{i}+0,745n_{m}-0.425n_{i}^{2}+0.024n_{m}^{2}-0.123n_{i}n_{m}\;$$

The model is statistically, although qualitatively, validated by the random distribution of the residuals as function of the employed variables and in a normal probability plot (both not shown for brevity). A quantitative validation is obtained by the analysis of variance (ANOVA) [27]. This procedure consists of calculating the sum of squares (SS) for the model and the error. When the relative degree of freedom (DF) is known, it is possible to calculate the mean square (MS) for the model and error. With the latter value, the F-value and the *p*-value can be determined for the model. The latter is a measure of the statistical significance of the model. In the present case, the very low *p*-value implies that the model is statistically significant (Table 2). 

**Figure 5:**
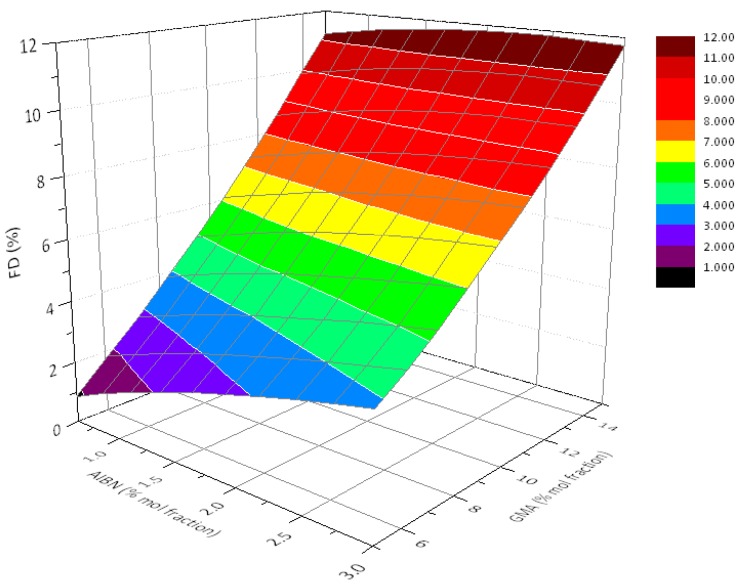
3D plot of model for FD from synthesis of PCL-*g*-GMA in scCO_2._

Furthermore, the obtained model can correctly describe the experimental data (as testified by the R^2^ and adjusted-R^2^ values) and has a very good predictive ability (R^2^-PRESS), at least within the range of variables studied here. The obtained model can be conveniently visualized in a 3D plot (Figure 5), which highlights the dependence of the FD values on the feed composition, as previously discussed. 

**Figure 6:**
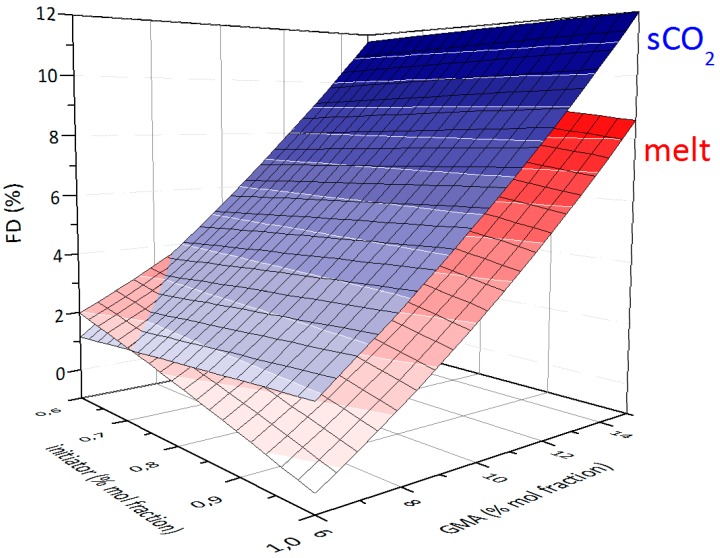
Comparison of grafting reactions in the melt and scCO_2._

Particularly interesting is the comparison between the process in the melt and the one in scCO_2_, which can be performed on the basis of the corresponding statistical models and 3D-plots (Figure 6) [25].

One should be aware that this comparison is limited to the intake values that were experimentally tested in both processes (initiator from 0.6% to 1% and GMA monomer from 6% to 15%). It might very well be that outside this range the behavior of these two reactions might be different that the one presented above. The use of two different initiators (BPO for melt, AIBN for scCO_2_) at two different temperatures might hinder a direct comparison. However, this has been easily overcome by employing appropriate reaction conditions, in particular a reaction time equal to 7 times the half-life time of the initiator at the given temperature (all initiator expected to be decomposed). In general, the scCO_2_ plot is always above the one for the melt process (except at low initiator and GMA intake) within the investigated range. The lack of data under scCO_2_ at relatively high GMA intakes (particularly above 15 mol %, see Table 1 as compared to Table S1) does not allow to extend this conclusion for higher FD values. Nevertheless, within the investigated range, this demonstrates the higher efficiency (in terms of FD values) for the grafting reaction in scCO_2_ as compared to the one in the melt. From a scientific point of view, a preliminary explanation for the observed trend might be related to the improved diffusion of GMA and initiator in scCO_2_ as compared to the melt.

### 3.2. Effect of Feed Composition on Molecular Weight

**Figure 7:**
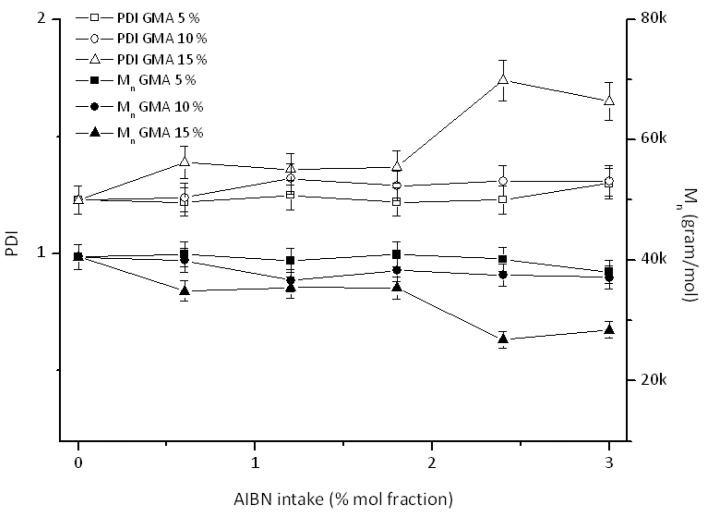
M_n_ and PDI as function of AIBN intake.

GPC analysis was performed to investigate the correlation between the functionalization degrees of the reactive interfacial agents and the corresponding molecular weight. Overall, the M_n_ values are generally constant (Figure 7).

This trend is in contrast to what reported by C-H. Kim et al., who observed an increase of the molecular weight probably related to chain extension [11]. In our case, no chain extension is observed, but a significant decrease (in *M*_n_), coupled to a broader distribution (PDI values), at high AIBN (2.4 and 3.0%) and GMA (15%) intakes. Such slight degradation is probably due to the rich radical environment, which could lead to scission. The *M*_n_ values are general a result of the two processes taking place here: grafting (and chain extension) on one side and degradation on the other. In the present case these two effects balance each other out at relatively low AIBN intakes while degradation seems to prevail for relatively higher AIBN concentrations.

**Figure 8:**
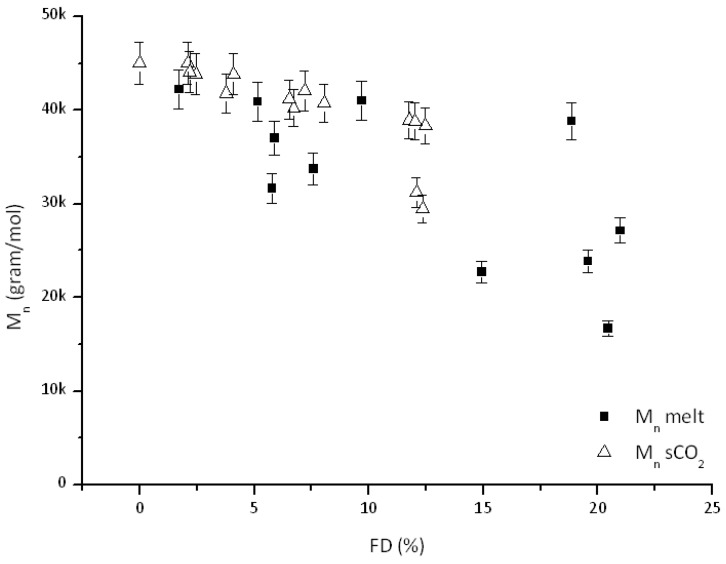
*M*_n_ comparison of reactive interfacial agents made in melt and scCO_2._

**Figure 9:**
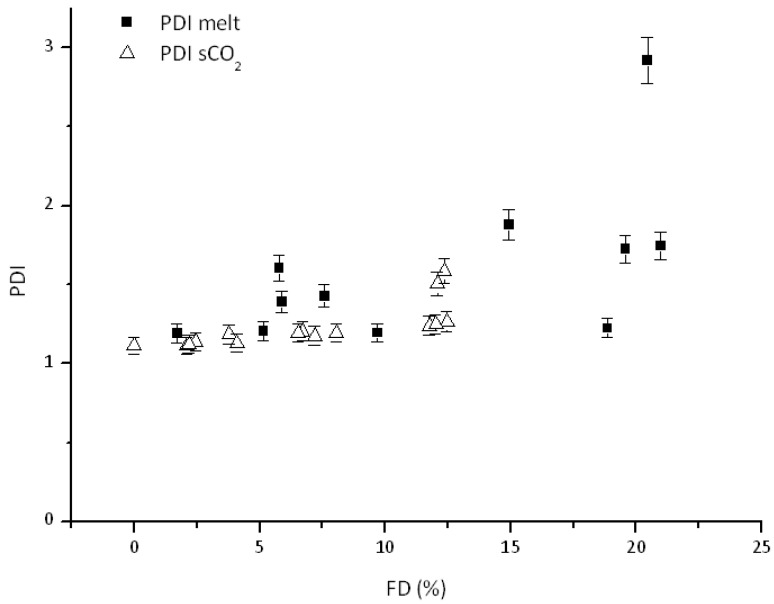
PDI comparison of reactive interfacial agents made in melt and scCO_2._

Also in this case, comparison with the corresponding data for process in the melt (Figure 8 and Figure 9) yields several interesting conclusions. 

In general, samples prepared in melt display only slight differences on observed M_n_ and larger PDI values compared to those processed using scCO_2_, see for example PDI values at about 6 mol % FD. This effect is even more relevant at relatively large FD values, even if such high FD values (above 12 mol %) were not investigated (nor attempted) under scCO_2_. This difference might then be caused by slower degradation reactions under scCO_2_ as already stated by several authors [17,21,22,26]. This might constitute a relevant issue when employing the prepared graft copolymers as interfacial agents in PCL-starch blends.

### 3.3. Thermal and Mechanical Properties for PCL-Starch Blends

The thermal properties of binary and ternary blends (PCL/starch/PCL-*g*-GMA) show a substantial invariance with respect to the GMA intake (on PCL-*g*-GMA) and starch content (See Supplementary Materials).

**Figure 10:**
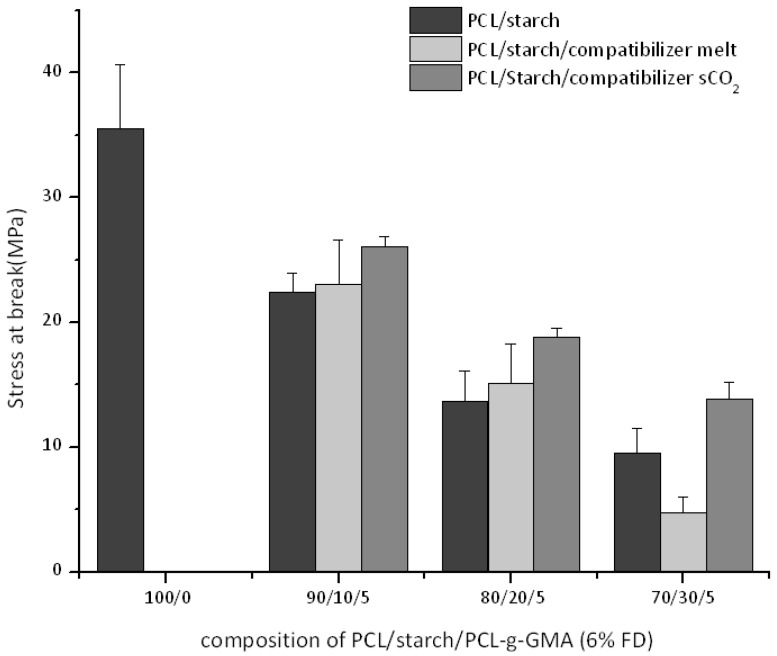
Stress at break for ternary PCL/Starch/PCL-*g*-GMA blends.

**Figure 11:**
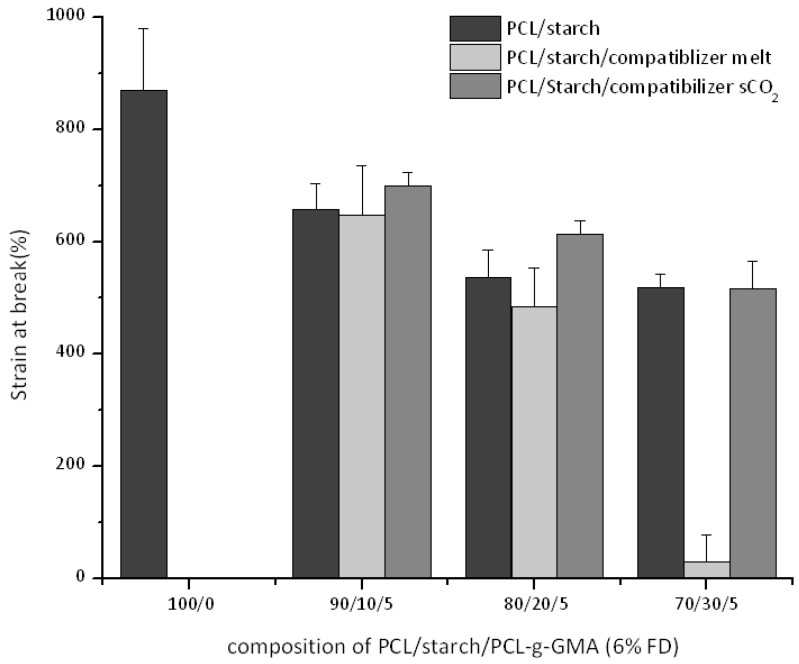
Strain at break for ternary PCL/Starch/PCL-*g*-GMA blends.

**Figure 12:**
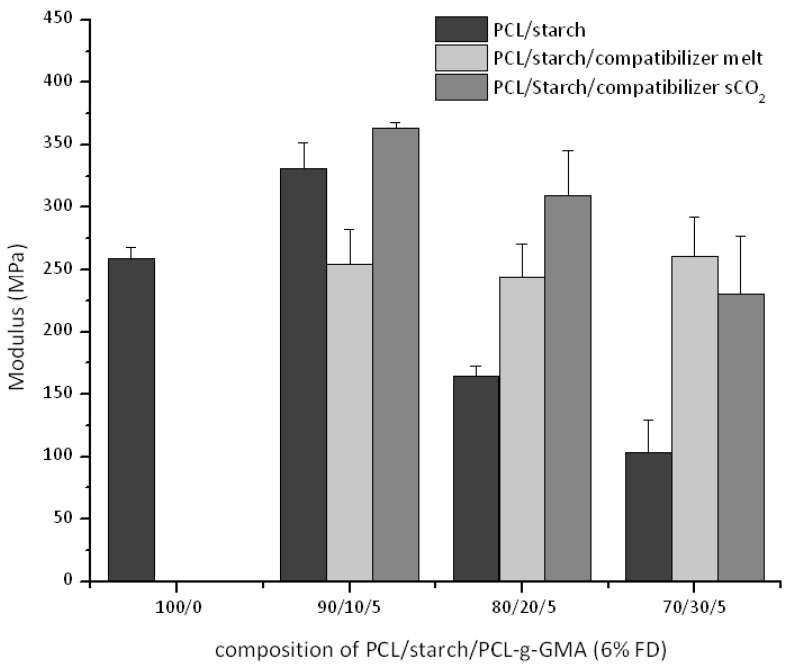
Modulus for ternary PCL/Starch/PCL-*g*-GMA blends.

The mechanical properties of the ternary blends (PCL/starch/PCL-*g*-GMA) are compared when using two interfacial agents with similar functionality (FD 6%) but prepared according to the two different processes (*vide supra*). We start noticing how the observed trends (Figure 10 and Figure 11) as function of the starch content (namely decrease in stress and strain at break) are in agreement with previous studies [12,13]. Compared to the binary blends, all compatibilized blends using interfacial agents prepared in scCO_2_ shows mechanical properties improvements (higher modulus and stress at break in Figure 10 and Figure 12). The strain at break remains factually the same (Figure 11).

On the other hand, blends with interfacial agents prepared in the melt show different trends. Equal stress and strain at break values (with respect to the corresponding binary blends) are observed at 10% and 20% starch intake while a lower value is detected at 30 wt %. Finally, the modulus of the blends is not affected by the starch addition and retains its value, similar to the one of virgin PCL. SEM images of all blends, independently of PCL-*g*-GMA the intake and the preparation methods (Figure S1), display factually the same morphology with a constant average starch particles size and distribution This is not surprising when making allowances for the fact that starch, used here without plasticizer, remains solid at the mixing temperature. One might speculate then that a higher PCL-*g*-GMA intake might result in a better interfacial adhesion (lack of voids at the interface in Figure S1). However, this is factually impossible to quantify based on the SEM images alone. 

A comparison of the mechanical properties as function of the employed process for the compatibilizer preparation (melt towards scCO_2_) shows that the stress and strain at break clearly decrease at starch intake of 30% (Figure 10 and Figure 11). The same is true for the modulus (Figure 12) except for the blends with 30 wt % starch (similar values). These results could be preliminarily explained by the (slight) difference in average molecular weight (Figure 8). However, it is debatable whether such small difference could be the cause for this dramatic improvement in mechanical properties. Another possible reason might lie in different topological characteristics for the two graft copolymers, as already suggested in our previous work [13]. To get deeper insight into the characteristics of the two graft copolymers (PCL-*g*-GMA from melt and scCO_2_), GPC measurements of the GMA homo-polymers (polyGMA) acquired from the purifications process of PCL-*g*-GMA were carried out. A clear difference in the degree of polymerization (DP) could be observed, with polyGMA from the scCO_2_ displaying shorter chains (scCO_2_-DP = 9 vs. melt-DP = 11). By assuming that the chain length of polyGMA grafted on the PCL backbone are proportional to those which are formed as by-product of the grafting process, one might speculate that at equal FD (as in this case) the PCL-*g*-GMA prepared in scCO_2_ has a larger amount of shorter GMA chains grafted onto the PCL backbone. This would in turn result in an easier (from a steric hindrance point of view) reaction of the GMA groups with the –OH ones on the surface of the starch particles [13]. Although speculative in nature, such hypothesis has also been formulated in connection to polymer blends comprising other polymeric components [28].

Independently of the exact mechanism and from a purely applicative point of view, one must stress here the superior, albeit slightly, performance of the scCO_2_-prepared compatibilizer with respect to its melt counterpart in terms of stress and strain at break at 30 wt % starch intake.

## 4. Conclusions

A series of reactive interfacial agents PCL-*g*-GMA has been synthesized under scCO_2_ and applied in ternary PCL/PCL-*g*-GMA/starch blends. The functionalization reaction in scCO_2_ clearly follows a different course with respect to the melt one. Higher FD values (at comparable feed composition) are generally obtained coupled with a substantial invariance (as opposed to thermal degradation in the melt) of average molecular weight and polydispersity of the starting PCL. However, the melt process makes it possible to prepare grafted polymers with higher FD values (in absolute terms) than the one in scCO_2_ under the employed experimental conditions. The scCO_2_-prepared interfacial agents also display a clear difference in performance when used in ternary blends with starch and PCL, significantly outscoring the melt prepared ones in terms of stress, strain at break and modulus at starch intakes of 30 wt %. This has been preliminarily explained in terms of the difference in molecular weight but also in topology (i.e., length and distribution of the grafted chains) of these graft copolymers. 

To the best of our knowledge this study offers for the first time a comprehensive overview of the advantages of scCO_2_ functionalization with the respect to the classical melt process. As such, it paves the way towards the definition and study of new functionalization reactions in supercritical media and application of the corresponding graft copolymers in industrially relevant products.

## Figures and Tables

**Table 1:** Overview of experiments for the PCL-*g*-GMA (T = 97 °C, P = 90 bar, t_reaction_ = 40 min).

Sample	Intake		PCL-*g*-GMA	
	GMA ^a^	AIBN ^a^	FD (%)	E (%)
PCL-*g*-GMA 1	5	0.6	0.0	0.0
PCL-*g*-GMA 2	5	1.2	2.5	46.2
PCL-*g*-GMA 3	5	1.8	2.1	39.1
PCL-*g*-GMA 4	10	0.6	4.1	36.6
PCL-*g*-GMA 5	10	1.2	6.7	59.7
PCL-*g*-GMA 6	10	1.8	7.2	63.5
PCL-*g*-GMA 7	15	0.6	12.5	70.1
PCL-*g*-GMA 8	15	1.2	11.8	65.7
PCL-*g*-GMA 9	15	1.8	12.0	66.6
PCL-*g*-GMA 10	5	2.4	2.2	40.4
PCL-*g*-GMA 11	10	2.4	6.5	57.2
PCL-*g*-GMA 12	15	2.4	12.7	68.1
PCL-*g*-GMA 13	5	3	3.8	69.6
PCL-*g*-GMA 14	10	3	8.1	70.1
PCL-*g*-GMA 15	15	3	12.1	66.0
PCL-*g*-GMA 16	8	1.2	6.0	68.1

^a^ mol fraction in percentage with respect to PCL

**Table 2:** Analysis of variance for FD model of PCL-*g*-GMA.

	SS	DF	MS	F-Value	*p*-Value	
Model	262.794	5	52.559	86.961	6.079 × 10^−7^	R^2^ = 0.978
Error	6.044	10	0.604			R^2^(adj) = 0.969
Total	268.838	15				R^2^(PRESS) = 0.933

## Supplementary Materials

The following are available online at http://www.mdpi.com/2073-4360/10/11/1285/s1, Table S1: Overview of experiments in the melt, Figure S1: Blends morphology as function of the PCL-*g*-GMA intake; Table S2: Selective solvent extraction data, Table S3: Thermal properties of the prepared blends.
