# Supplementary material for: Green Processes for Green Products: The Use of Supercritical CO2 as Green Solvent for Compatibilized Polymer Blends
Source: Polymers (Basel). 2018 Nov 19;10(11):1285. doi: 10.3390/polym10111285 (PMC6401873; doi:10.3390/polym10111285)
Supplement: Supplementary file 1 [file polymers-10-01285-s001.zip › polymers-370218-SI.pdf]

Polymers 2018, 10, 1285; doi:10.3390/polym10111285

Supplementary information

Table S1 Overview of experiments in the melt

Sample	Intake (%-mol)			FD (%)
	GMA	BPO	PCL	
**PCL-g-GMA 1**	12	0.60	87.40	5.89
**PCL-g-GMA 2**	24	0.60	75.40	20.98
**PCL-g-GMA 3**	36	0.60	63.40	45.62
**PCL-g-GMA 4**	6	0.30	93.70	1.71
**PCL-g-GMA 5**	24	1.10	74.90	20.45
**PCL-g-GMA 6**	12	0.30	87.70	7.58
**PCL-g-GMA 7**	10	0.30	89.70	5.16
**PCL-g-GMA 8**	18	0.30	81.70	14.93
**PCL-g-GMA 9**	24	0.30	75.70	19.58
**PCL-g-GMA 10**	12	1.10	86.90	5.78
**PCL-g-GMA 11**	36	1.10	62.90	36.21
**PCL-g-GMA 12**	12	0.10	87.90	9.69
**PCL-g-GMA 13**	24	0.10	75.90	18.86

Morphology characterization

The morphology of the investigated blends was studied by SEM, for example as function of the compatiblizer (PCL-g-GMA) intake (Figure S1).

Figure S1. Blends morphology as function of the PCL-g-GMA intake.

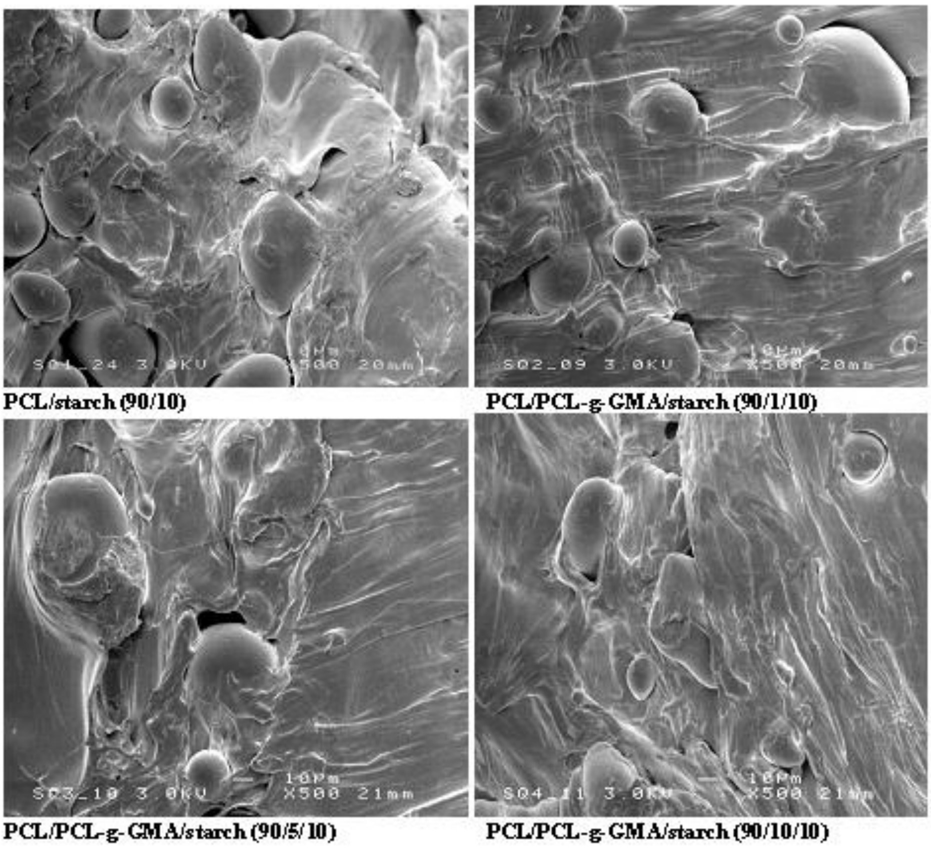

While a clear difference in the interfacial adhesion (lack of voids) can be seen between the blend without PCL-g-GMA and the rest (especially at 10 wt % intake), no clear trends can be detected.

Selective solvent extraction

Extraction with chloroform was used to characterize, albeit indirectly, the reaction at the interface between PCL-g-GMA and starch. To this end a blend PCL-g-GMA/S 70/30 was prepared according to the same procedure as for all the others and extracted together with the corresponding reference PCL/S (Table S???).

Table S2. Selective solvent extraction data

Sample[a]	Insoluble fraction (wt %)
PCL/S 70/30	29±2
PCL-g-GMA/S 70/30	32±2

[a] Samples are denoted by their components followed by their weight fractions

Although the measured values are quite close to each other, their difference indicates that a part of PCL-g-GMA (contrary to PCL) is retained in the insoluble fraction.

DSC characterization

Blends were also characterized by DSC analysis (Table S3). The results show a substantial invariance of the thermal properties as function of the GMA intake (see data for PCL/S/PCL-g-GMA 80/20/5). It is worth noticing here that at FD=15 mol % an increase in the PCL melting and crystallization enthalpy is detected and probably due to a nucleation effect of the GMA groups.

Table S3. Thermal properties of the prepared blends.

**Sample**	**FD (mol %) PCL-g-GMA**	**T_c (°C)**	**ΔH_c (J/g_{PCL})**	**T_m (°C)**	**ΔH_m (J/g_{PCL})**
PCL/S 100/0	n.a.	33	55	53	68
PCL/S 90/10	n.a.	34	51	57	69
PCL/S 80/20	n.a.	32	50	56	75
PCL/S 70/30	n.a.	32	47	57	66
PCL/S/PCL-g-GMA 90/10/1	6	33	56	56	75
PCL/S/PCL-g-GMA 90/10/5	6	35	53	54	74
PCL/ S/PCL-g-GMA 90/10/10	6	34	57	57	74
PCL/ S/PCL-g-GMA 80/20/5	15	33	63	56	87
PCL/ S/PCL-g-GMA 80/20/5	6	33	56	56	74
PCL/ S/PCL-g-GMA 80/20/5	2	34	57	55	77
PCL/ S/PCL-g-GMA 70/30/5	6	33	51	56	68

On the other hand, the presence of starch has a clear influence on the thermal properties and namely resulting in an increase of the PCL melting and crystallization enthalpies, probably due to a similar effect as the one proposed above.
